# Human myiasis in Sub-Saharan Africa: A systematic review

**DOI:** 10.1371/journal.pntd.0012027

**Published:** 2024-03-28

**Authors:** Binta J. J. Jallow, Goudja Gassara, Ousman Bajinka, Yifei Luo, Mandie Liu, Jifeng Cai, Jingjing Huang, Fanming Meng

**Affiliations:** 1 Central South University, Department of Medical Parasitology, Changsha City, China; 2 Central South University, Department of Nutrition Science and Food Hygiene, Xiangya School of Public Health, Changsha City, China; 3 University of The Gambia, School of Medicine and Allied Health Science, Banjul City, Gambia; 4 Xinjiang Medical University, Department of Forensic Medicine, School of Basic Medical Sciences, Urumqi City, China; University of Queensland & CSIRO Biosecurity Flagship, AUSTRALIA

## Abstract

**Background:**

Human myiasis is a parasitic dipteran fly infestation that infects humans and vertebrates worldwide. However, the disease is endemic in Sub-Saharan Africa and Latin America. In Sub-Saharan Africa, it is under-reported and therefore its prevalence is unknown. This systematic review aims to elucidate the prevalence of human myiasis, factors that influence the infection, and myiasis-causing fly species in SSA. The review also dwelled on the common myiasis types and treatment methods of human myiasis.

**Methods:**

Here, we collect cases of human myiasis in Sub-Saharan Africa based on literature retrieved from PubMed, Google Scholar and Science Direct from 1959 to 2022. A total of 75 articles and 157 cases were included in the study. The recommendations of PRISMA 2020 were used for the realization of this systematic review.

**Results:**

In total, 157 cases of human myiasis in SSA were reviewed. Eleven fly species (*Cordylobia anthropophaga*, *Cordylobia rodhaini*, *Dermatobia hominis*, *Lucilia cuprina*, *Lucilia sericata*, *Oestrus ovis*, *Sarcophaga* spp., *Sarcophaga nodosa*, *Chrysomya megacephala*, *Chrysomya chloropyga* and *Clogmia albipuntum*) were found to cause human myiasis in SSA. *Cordylobia anthropophaga* was the most prevalent myiasis-causing species of the reported cases (n = 104, 66.2%). More than half of the reported cases were from travelers returning from SSA (n = 122, 77.7%). Cutaneous myiasis was the most common clinical presentation of the disease (n = 86, 54.7%). Females were more infected (n = 78, 49.6%) than males, and there was a higher infestation in adults than young children.

**Conclusion:**

The findings of this study reveals that international travelers to Sub-Saharan Africa were mostly infested therefore, we recommend that both international travelers and natives of SSA be enlightened by public health officers about the disease and its risk factors at entry points in SSA and the community level respectively. Clinicians in Sub-Saharan Africa often misdiagnose the disease and most of them lack the expertise to properly identify larvae, so we recommend the extensive use of molecular identification methods instead.

## Author summary

Human myiasis is a neglected tropical disease in the world especially in SSA. Human myiasis in SSA has infects patients from all dynamics especially those with underlying health issues like primary wounds. The disease can be fatal especially when it involves heavy infestation of the scalp (migratory myiasis) among young children. Subcutaneous myiasis of the eye and genitals can be devastating for patients and can lead to damage in these areas if the disease is misdiagnosed and larvae removal is delayed. The findings and recommendations in our study can be used by government officials in SSA to provide hospitals with state-of-the-art diagnostic tools, trained entomologists, and research funding to comprehensively study human myiasis. Public health officers at entry points in SSA should inform international travelers about the risk factors of the disease and common preventive measures. Clinicians in SSA should report more human myiasis cases to enable researchers to estimate the epidemiology and prevalence of the disease. The natives of SSA should be enlighten more on the symptoms and risk factors of the disease and encourage them to report to health facilities when they experience these symptoms.

## 1. Introduction

Myiasis, coined from the Greek word ‘myia’ meaning fly, is the infestation of live or dead tissues of vertebrates (humans and animals) by immature stages (maggots) of dipteran flies [1,2]. The disease dates back to 1840 when it was first described by Hope [3] and is still considered a neglected disease in humans, especially in the tropical and sub-tropical regions in SSA, Asia, and Latin America [4]. The disease has a worldwide distribution and has been endemic in Latin America and SSA for years. However, with the increase in global travel, the disease has spread widely, especially in areas with warmer temperatures and high humidity [5]. Myiasis is more common in animals, such as sheep, rodents, and antelope, than humans because humans are accidental hosts. Furuncular myiasis is the most common myiasis reported from travelers returning from endemic regions and is usually caused by the human botfly, *Dermatobia hominis* in Latin America. In SSA, the tumbu fly or mango fly (*Cordylobia anthropophaga*) causes year-round infestation which could be dated back to 1904 [6], albeit most of the human myiasis infestation in SSA are caused by this species [7]. The climate condition in SSA is suitable for the breeding of some fly species which makes most places to be endemic of them. Although human myiasis is endemic in SSA, the diversity and prevalence of myiasis-causing flies in SSA is still not clear to date.

Human myiasis can be categorized depending on several factors. According to the host-parasitic relationship (feeding relationship between larva and the host), myiasis can be divided into obligatory myiasis, facultative myiasis, and accidental myiasis. In obligatory myiasis, fly larvae require living tissues for survival and to complete the immature stages of their life cycle. Facultative myiasis on the other hand is caused by free-living fly species (feeding on decaying organic matter and can opportunistically infest living tissues), their larvae do not require a living host to complete their life cycle. While accidental myiasis is a condition in which the larval stages of dipteran flies are accidentally ingested through contaminated food or water [8,9]. Additionally, human myiasis can further be classified into primary and secondary myiasis. When dipteran fly larvae invade healthy tissues or skin it will result in primary myiasis, and when these larvae colonize pre-existing wounds it will result in secondary myiasis [4,10].

**Fig 1:**
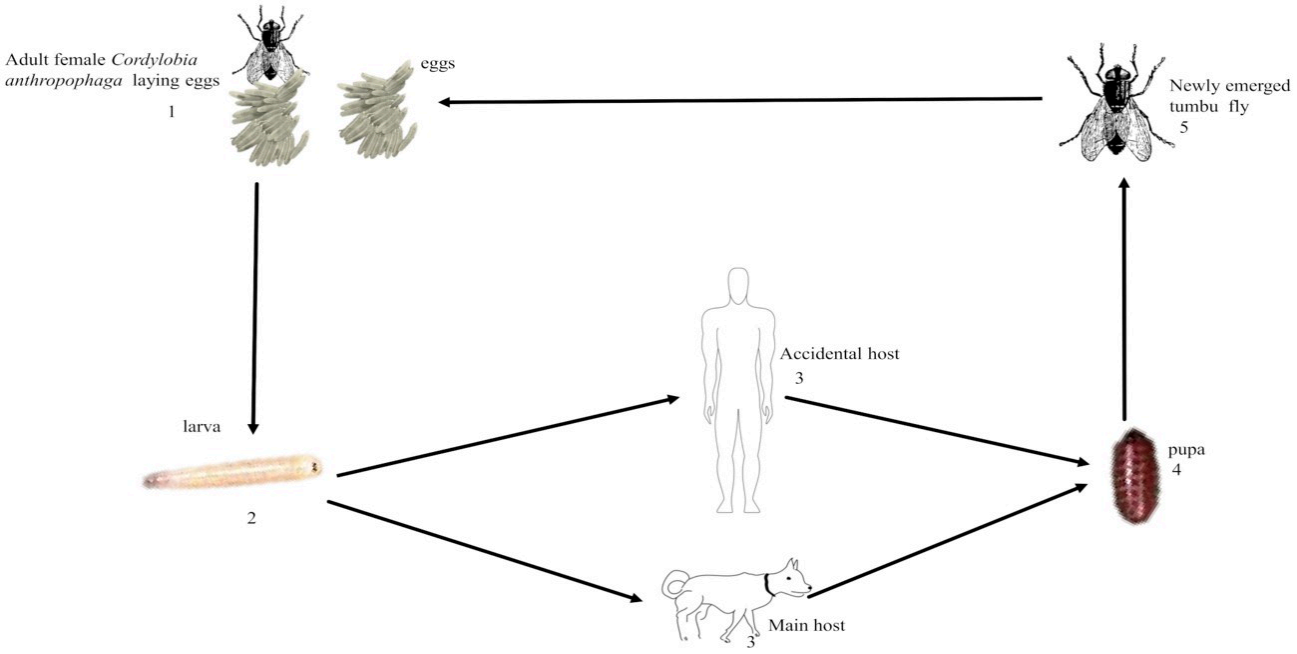
Life cycle of *Cordylobia anthropophaga*. **1.** Adult female *Cordylobia anthropophaga* lay 100–300 eggs on the on wet clothing or faecal-contaminated soil. **2.** Eggs catch to form 1^st^ instar larva d. **3.** 1^st^ instar larva penetrates a host which is usually dog, or rodent, but could accidently penetrate a human host and develop to 3^rd^ instar larva. **4.** Larva leaves its host to the ground to pupate. **5.** pupa metamorphosis to an adult fly.

According to the anatomical site or clinical presentation, myiasis can be cutaneous which involves the infestation of dermal and sub-dermal layers (tissues) of the skin (humans and animals) or infest any part of the body (nose, eyes, scalp, breast, intestine, leg, urogenital, mouth, arms, and thighs) [11]. Cutaneous myiasis takes account the largest part of clinical presentation in humans which could be categorized into migratory (creeping) myiasis, furuncular myiasis, and wound (traumatic) myiasis [Fig 1] [12]. Similarly, furuncular myiasis is the most common type reported from travelers from endemic regions, and is characterized by the formation of a painful inflammatory nodule with a central punctum on healthy or unbroken skin [1]. It is mainly caused by the tumbu fly or the botfly [13]. Wound (traumatic) myiasis is caused by dipteran fly larvae which colonize pre-existing wounds and enlarge them [8,10]. While migratory (creeping) myiasis is a condition in which dipteran fly larvae burrow in the subcutaneous tissues of the host and migrate, and often causes pruritic lesions within the host tissues. Theppote A., et al., 2020 presented a clinical presentation of cutaneous myiasis [12].

Compelling scientific literature have revealed that a significant rise in temperature or humidity will increase the growth and redistribution of most myiasis-causing flies, subsequently increasing myiasis infestation in such regions [11,14]. Some factors that influence this dermatosis in SSA are high humidity and temperature (especially during the rainy season) [5]. Poverty, and poor hygiene also add to the vulnerability to human myiasis infestation. Rodents, antelopes, and pet animals, especially dogs, are the hosts of tumbu fly, making farmers, and pet keepers more vulnerable to *C*. *anthropophaga* infestation [15,16]. Skin-related diseases belong to the leading travel-related health problems reported. Currently, human myiasis is reported as one of the five most common travel-related skin diseases, which accounts for 7–12% of travel-related diseases globally [1,17]. Hence, an increase in international travel will drastically increase human myiasis infestation in both endemic and non-endemic regions.

Human myiasis can affect people (tourists, businesspersons, etc.) traveling to endemic regions, especially in SSA, where it remains a burden and needs urgent attention. Both travelers and natives of SSA lack awareness of how to prevent themselves against human myiasis infestation which adds to the vulnerability of the disease. Wearing long garments to cover legs and hands, especially during the rainy season, and sleeping in bed nets would help prevent insect bites. Lying on the ground, hanging clothes on shady lines or bushes, and lack of ironing of garments or bedding after laundry should be avoided [17]. The use of some fly repellents is encouraged to avoid insect bites. Open wounds should be routinely dressed, good skincare and standard hygiene should be maintained to avoid human myiasis infestation [16]. Animal pets should be properly handled because some of these pets can be reservoirs for the disease and can add to the vulnerability of human myiasis infestation.

This parasitological condition causes harm not only to humans but also to the livestock industry, accruing substantial economic losses for farmers [8,18]. Human myiasis is often misdiagnosed as cellulitis, leishmaniasis, tungiasis, or furunculosis [19] which is a common problem in diagnosing the disease. Therefore, properly extracting fly maggots, and sometimes the use of antibiotics becomes the gold standard for the treatment of human myiasis cases. Morphological identification is considered standard for larval identification in human myiasis cases, however, the use of molecular identification method has been being utilized globally. This method can differentiate closely related species and identify immature stages which could be an effective method in cases where traditional morphological method is ineffective [1,20,21]. Secondary bacterial superinfection and tetanus are some of the severe complications of human myiasis especially if larvae fragmentation occurs during removal [16,21,22]. Subsequently, the need to systematically review the current literature on human myiasis in SSA is an important priority. This review aims to elucidate the prevalence of human myiasis, and highlight the most common myiasis-causing flies and areas where they are endemic in SSA. We wish to uncover the predisposing factors of human myiasis in SSA and highlight the most common clinical forms of myiasis. Our study will equally highlight the common extraction and treatment forms of human myiasis.

### Ecology and Life Cycle of *Cordylobia anthropophaga*

The tumbu fly is the most common myiasis-causing fly in SSA. The adult tumbu fly is yellowish-brown in colour measuring 6–12 mm in length with two bands on the thoracic region and a brownish-black on the abdomen [23]. The female tumbu fly lays approximately 100–300 eggs on urine and faecal-contaminated soil or wet clothing (linens), especially clothes dried on shady lines and or bushes which are favourable oviposition locations [11]. When eggs hatch, the first instar larvae penetrate the host skin [24] and after 7–12 days, the second and third instar larvae of tumbu fly will be formed. These stages are characterized by a cuticular spine, spiracular plates, and peritrenes on both the anterior and posterior ends. The third instar larva of *C*. *anthropophaga* leaves the host to the ground to pupate and becomes an adult fly and this cycle is repeated [Fig 1] [25].

## 2. Materials and methods

### 2.1 Search strategy

According to the PRISMA Recommendations (Preferred Reporting Items for Systematic Reviews and Meta-Analyses) of 2020 [26], a systematic review of the literature was carried out in pairs. A literature search was conducted in Science Direct, PubMed, and Google scholar using the following search terms: human, myiasis, Sub-Saharan Africa (SSA), and case reports. Boolean operators (AND, OR) were used to combine search terms. We searched manually using the references of retrieved articles and thereby identified articles that were not retrieved from the database search. The search focused on studies conducted in all (48) countries in SSA and the search covered the years between 1959 and 2022 with no filter applied. Case reports from countries in SSA or those acquired from travel to SSA were considered. The following combined keywords were used for the search:

1. For PubMed, search terms were: human myiasis AND SSA AND case reports;
2. For Google Scholar, the search terms used were: human myiasis AND SSA AND case reports.
3. For Science Direct, search terms were: human myiasis AND SSA AND case reports.

It was worth noting that papers were also retrieved using the search terms; human myiasis AND country name AND case reports for all countries in SSA and this was done for more thorough search.

### 2.2 Inclusion and exclusion criteria

The inclusion criteria were: (1) original articles related to the topic of interest of this study; (2) any case report article from SSA with no language restriction and/or could be translated using Google translate; (3) relevant case reports from SSA found in review studies; (4) studies carried out in SSA; (5) studies published between 1959 to 2022.

The exclusion criteria of this were: (1) articles that are not case reports and articles with case reports that are not from SSA; (2) articles with no author’s name and year of publication; (3) review studies that are with no relevant case reports; (4) unpublished studies; and (5) non-human studies.

### 2.3 Data extraction

All duplicate articles were removed using the Endnote library. Three separate examiners carried out the first phase of the selection. It allowed us to delete certain selected studies. Thus, after a complete reading, we selected the studies that met the inclusion criteria. Discrepancies were resolved by discussion with a fourth reviewer. The following information was extracted from the articles selected; patient’s age, sex, country of origin, myiasis type, site of infection, country reported, number of patients, and country of infestation [Table 1]. The studies will be grouped according to the sub-regions SSA (West Africa, East Africa, Southern Africa, and Central Africa) based on the United Nations (UN) system classification. A protocol for this study has been upload on protocol.oi with a DOI of DOI: dx.doi.org/10.17504/.

**Table 1:** Summary of selected studies.

**Central Africa**								
**Myiasis type**	**Patient, Age**	**Sex**	**Fly species**	**Site of infection**	**Country reported**	**No. of patients**	**Country of infestation**	**Ref**
Furuncular	29 (average)	M	*C*. *anthropophaga*	Various body parts	Central Africa	16	Local infestation	[27]
Furuncular	59	F	*C*. *rodhaini*	Scalps	France	1	Cameroon	[28]
Furuncular	50	M	*C*. *anthropophaga*	Thorax, back, lower lip	Vienna	1	Cameroon	[29]
Furuncular	46	F	*C*. *anthropophaga*	Right lumbar, gluteal region	Panama	1	Central Africa	[30]
Cutaneous	55	M	*C*. *anthropophaga*	Left buttock upper thigh	Korea	1	Central Africa	[23]
Cutaneous	46	M	*C*. *anthropophaga*	Anterior chest a acromioclavicular joint	Sri-lanka	1	Cameroon	[31]
Cutaneous	54	M	*C*. *anthropophaga*	Entire back	Britain	1	Congo	[32]
Glans (penis)	22	M	*C*. *anthropophaga*	Near the opening of urethra	Congo	1	Local infestation	[33]
Furuncular	5 months	M	*C*. *anthropophaga*	Right shoulder	France	1	Congo	[34]
Furuncular	61	F	*C*. *anthropophaga*	sub clavicular	Italy	1	Cameroon	[35]
Furuncular	37	M	*C*. *anthropophaga*	Chest & shoulder	Morocco	1	Congo	[36]
Furuncular	59	F	*C*. *rodhaini*	Scalp, left parietal temporal part, right flank, upper lip intranasal lesion and abdomen	Morocco	1	Cameroon	[37]
Cutaneous	-	-	*D*. *hominis*	leg	Morocco	1	Congo	[38]
**East Africa**								
**Myiasis type**	**Patient Age**	**Sex**	**Fly species**	**Site of infection**	**Country reported**	**No. of patients**	**Country of infestation**	**Ref**
Furuncular	45	F	*C*. *anthropophaga*	Left thigh	Sudan	1	Local infestation	[39]
Cutaneous	31, 28	M, F	*C*. *anthropophaga*	Trunk penis lower leg, left buttock	Germany	2	East Africa	[40]
Furuncular	21	F	*C*. *anthropophaga*	Left finger	Japan	1	Uganda	[41]
Cutaneous	46	F	*C*. *anthropophaga*	Upper right arm	Sudan	1	Local infestation	[42]
Furuncular	61	F	*C*. *anthropophaga*	Left arm, abdomen, left, thigh	Ethiopia	1	Local infestation	[43]
Furuncular	59, 58	M, F	*C*. *rodhaini*	Left shoulder, abdomen Pubis	Italy	2	Uganda,	[13]
Furuncular	52	F	*C*. *rodhaini*	Right leg	Italy	1	Ethiopia	[13]
Furuncular	42,5	M, M	*C*. *rodhaini*	Left buttock, top right side of head & maxilla	Ethiopia	2	Local infestation	[44]
Furuncular	22	F	*C*. *anthropophaga*	Right gluteus	Italy	1	Kenya	[17]
Furuncular	56	-	*C*. *anthropophaga*	Upper thigh, lower abdomen, lower back	USA	1	Ethiopia	[45]
Ocular	59	M	*C*. *anthropophaga*	Right eye lid	Italy	1	Kenya	[46]
Furuncular	-	M	*C*. *rodhaini*	Scalp, right arm, left arm, torso	Italy	1	Uganda	[8]
Furuncular	33	M	*C*. *anthropophaga*	Left leg	Korea	1	Uganda	[1]
Furuncular	-	-	*C*. *anthropophaga*	Left thigh	-	1	Tanzania	[47]
Cutaneous	30	M	*C*. *anthropophaga*	Left groin	UK	1	Sierra Leone	[48]
Furuncular	57	F	*C*. *rodhaini*	Right thigh	Australia	1	East Africa	[49]
Furuncular	-	M	*C*. *rodhaini*	All over the body	Italy	1	Ethiopia	[50]
Cutaneous	52	F	*C*. *rodhaini*	-	Germany	1	Tanzania	[51]
Cutaneous	38	M	*C*. *anthropophaga*	Left lower back	China	1	Ethiopia	[52]
Cutaneous	55	F	*C*. *rodhaini*	Fore head	UK	1	Uganda	[53]
Cutaneous	26	F	*C*. *anthropophaga*	Left upper arm	China	1	Uganda	[20]
Cutaneous	26	F	*C*. *rodhaini*	Left upper arm	Canada	1	Ethiopia	[54]
Furuncular	4month, 13month	F, -	*C*. *anthropophaga*	Aural flanks, chin, and fore legs, Lower trunk, forearm and right arm	Ireland	2	Kenya	[55]
Furuncular	26	M	*C*. *anthropophaga*	Fifth digit on foot	USA	1	Tanzania	[56]
Cutaneous	26	M	*C*. *anthropophaga*	Back (mid scapular region)	UK	1	Kenya	[57]
**West Africa**								
**Myiasis type**	**Patient Age**	**Sex**	**Fly species**	**Site of infection**	**Country reported**	**No. of patients**	**Country of infestation**	**Ref**
Cutaneous	17	F	*C*. *rodhaini*	Left breast	UK	1	Ghana	[58]
Furuncular	42	F	*C*. *anthropophaga*	Left buttocks	France	1	Senegal	[28]
Glans penis	10	M	*C*. *anthropophaga*	penis	Denmark	1	Senegal	[59]
Cutaneous	-	F	*C*. *anthropophaga*	Breast, upper & lower lips	Nigeria	28	Local infestation	[60]
Furuncular	45	M	*C*. *anthropophaga*	Limbs & trunk	India	1	Nigeria	[61]
Cutaneous	29	F	*C*. *anthropophaga*	-	US	1	Sierra Leone	[62]
Furuncular	-	F	*C*. *rodhaini*	On the thigh, below the breast	Israel	2	Ghana	[63]
Furuncular	16, 17	M, F	*C*. *anthropophaga*	Bilateral legs, ankle, left thigh, buttocks	USA	2	Senegal	[64]
Cutaneous	48, 47, 14	M, F, F	*C*. *anthropophaga*	Back & nose, shoulder, wrist, back	Slovenia	3	Ghana	[65]
Furuncular	32	F	*C*. *anthropophaga*	Thigh, left flank	Britain	1	Gambia	[66]
Cutaneous	30	F	*C*. *anthropophaga*	Right leg	Italy	1	Senegal	[67]
Furuncular	45		*C*. *anthropophaga*	Aural flanks, chin, and fore legs	Vienna	1	Senegal	[68]
Cutaneous	6 weeks	Child	*C*. *anthropophaga*	Scalp, dorsal part of trunk, hands, leg	Gambia	1	Local infestation	[11]
Vulva	16	F	*C*. *anthropophaga*	vulva	-	1	Senegal	[69]
Cutaneous	50	M	*C*. *anthropophaga*	Sole of the left foot	Italy	1	Senegal	[70]
Furuncular	-	-	*D*. *hominis*	-	Nigeria	1	Local infestation	[71]
furuncular	29	F	*D*. *hominis*	buttocks	Spain	1	Guinea Bissau	[72]
Ocular	24	F	*C*. *anthropophaga*	Upper eye lid	France	1	Cape Verde	[73]
Furuncular	12	F	*C*. *anthropophaga*	Lower lid of the right eye and forearm	Thailand	1	Ghana	[74]
Cutaneous	55	M	*C*. *anthropophaga*	Trunks, arms and legs	USA	1	Nigeria	[22]
Nasopharyngeal	Adult	M	*Clogmia albipunctatum*	Nose	UK	1	Nigeria	[75]
Cutaneous	27	M	*C*. *anthropophaga*	-	Netherlands	1	Gambia	[76]
Furuncular	11month, 10y	-, F	*C*. *anthropophaga*	Right arm, thigh	Nigeria	2	Local infestation	[77]
Cutaneous	52	M	*D*. *hominis*	Upper lip	UK	1	Gambia	[78]
Furuncular	30	F	*C*. *anthropophaga*	Lower back	USA	1	Guinea	[79]
Cutaneous	70	F	*C*. *anthropophaga*	Right breast	Nigeria	1	Local infestation	[80]
Cutaneous	24, 23	F, F	*C*. *anthropophaga*	Left flank, right thigh	Italy	2	Senegal	[81]
Cutaneous	34, 21	F, F	*C*. *anthropophaga*	Breast, breast	Nigeria	2	Local infestation	[60]
**South Africa**								
**Myiasis type**	**Patient Age**	**Sex**	**Fly species**	**Site of infection**	**Country reported**	**No. of patients**	**Country of infestation**	**Ref**
-	22 &57	-	*Sarcophaga spp & L*. *cuprina*	Various body part	South Africa	2	Local infestation	[82]
Ocular	10	M	*Oestrus ovis*	Right and left eye	India	1	South Africa	[83]
-	-	Infants	*C*. *anthropophaga*	Arms, buttocks, trunk	South Africa	6	-	[84]
Ocular	27	F	*Oestrus ovis*	eyes	Greece	1	South Africa	[85]
Furuncular	6-month	F	*C*. *anthropophaga*	Thighs, trunk	Malawi	1	Local infestation	[19]
Furuncular	39	M	*C*. *anthropophaga*	Left eye, right thigh, left buttocks	UK	1	Angola	[86]
Wound or traumatic	51	M	*L*. *cuprina*	foot	South Africa	1	Local infestation	[87]
Cutaneous	28	F	*C*. *anthropophaga*	Upper arms, wrist & inner thigh	Australia	1	South Africa	[88]
Furuncular	57	F	*-*	Left shoulder, back, left arm, right thigh, popliteal fossa	UK	1	South Africa	[89]
Furuncular	38	M	*C*. *anthropophaga*	Right side of his scalp	UK	1	South Africa	[90]
Cutaneous	56 (average)	M(14), F(11)	*L*. *cuprina*, *L*. *sericata*, *C*.*megacephala*, *C*. *chloropyga*, *Sarcophaga*, *2UI*	Different body parts	South Africa	25	Local infestation	[91]

### 2.4 Quality assessment

The risk of bias was assessed by the Joanna Briggs Institute (JBI) checklist for Case Reports Critical Appraisal Tool [92]. Two reviewers independently assessed selected articles, and discrepancies were resolved by discussion or by the other reviewers. The assessment of the quality of the selected studies is presented in the S1 Table. The selected studies were homogeneous, and 73 of 75 studies were of high quality, one study was moderate and one study was below quality according to the JBI-MAStARI.

## 3. Results

**Fig 2:**
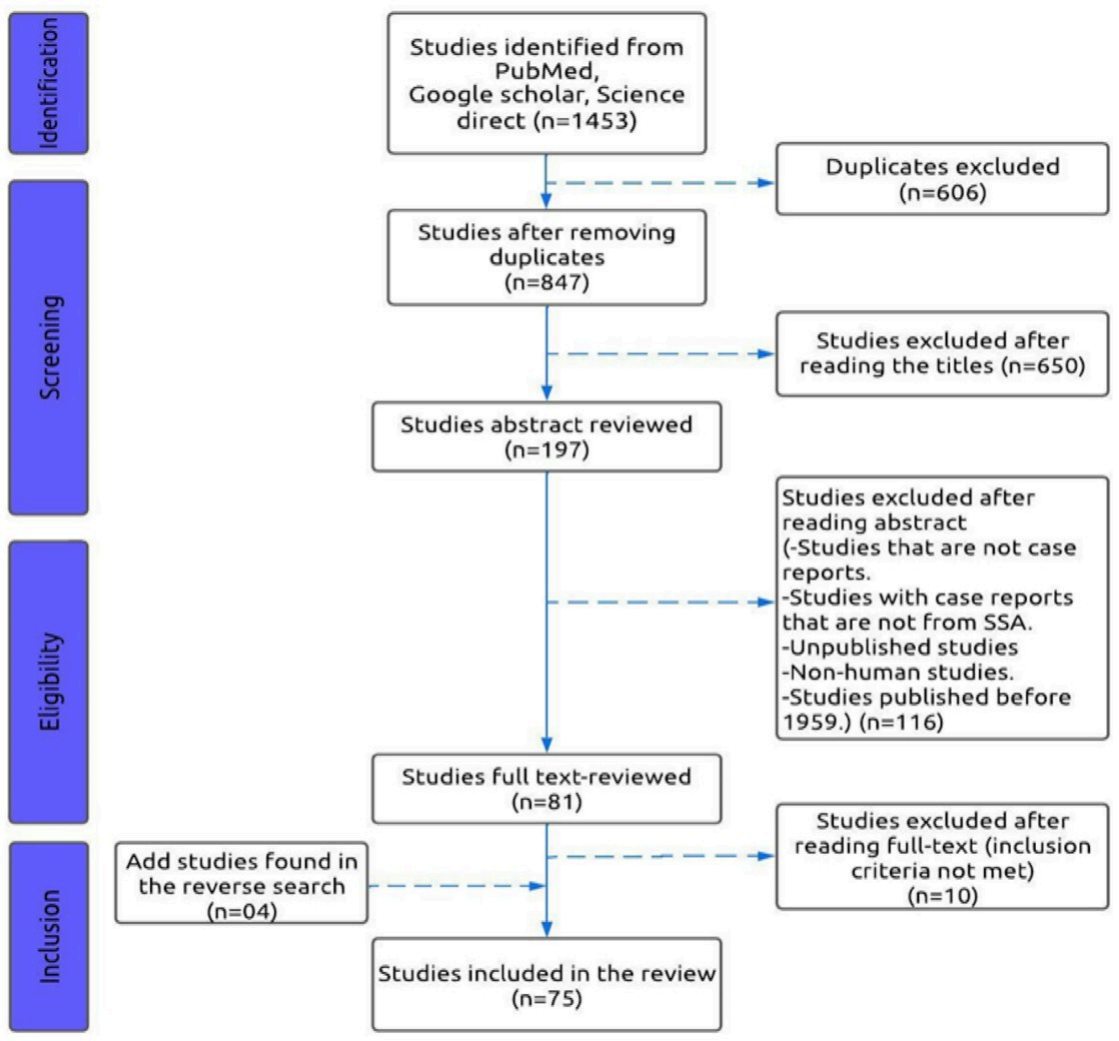
Flowchart for the selection of studies based on the PRISMA 2020 guidelines.

There were 1,453 original articles identified in the three databases, of which 847 articles were retained after the deletion of duplicates. A selection based on title led to the exclusion of 650 articles. Abstracts of the remaining 197 articles were reviewed, excluding 116 more articles. Reading the full text of the remaining 81 articles allowed the exclusion of 10 articles. Finally, 71 articles which met the inclusion criteria were selected. A reverse search was performed on the 71 included articles by searching the terms in the references of the selected articles to identify the articles which had not been initially selected and which fulfilled the inclusion criteria. Thus, other articles were identified and included, totaling 75 articles for this systematic review. The representative search design and number of eligible studies are shown in Fig 2.

### 3.1. Characteristics of included studies

Table 1 summarized all included studies in this systematic review and were grouped by sub-regions. Thus, 13 studies were identified from Central Africa, 23 in East Africa, 28 in West Africa, and 11 in Southern Africa. There were 75 studies in total and the cases from these study included both males and females of different age groups. Some cases do not contain gender information. There were 7 classifications of human myiasis (cutaneous, furuncular, ocular, glancpenis, vulva, wound, and nasopharyngeal myiasis) detected in this study. The sample size ranged from 1 to 157 patients, and patients’ ages ranged from 6 weeks to 70 years (the average age is 17 years).

### 3.2. Evidence from reviewed studies

Table 1 summarizes the different types of myiasis, fly species, and site of infection. This table also shows the countries where these infections were reported, the number of patients, and the country of infestation.

Regarding myiasis types, cutaneous myiasis was described by more than half (54.7%) of the studies examined (n = 86). Furuncular myiasis was described by 38.2% of the articles retained (n = 60). Three (n = 03) articles reviewed genital myiasis (glans penis, and vulva) representing 1.2% and 0.6%, respectively. Very few studies (n = 4) described ocular myiasis (2.5%). In addition, two (n = 2) (1.2%) studies reviewed had described nasopharyngeal myiasis and wound myiasis. There were 2 (1.2%) cases with un-identified myiasis type.

**Fig 3:**
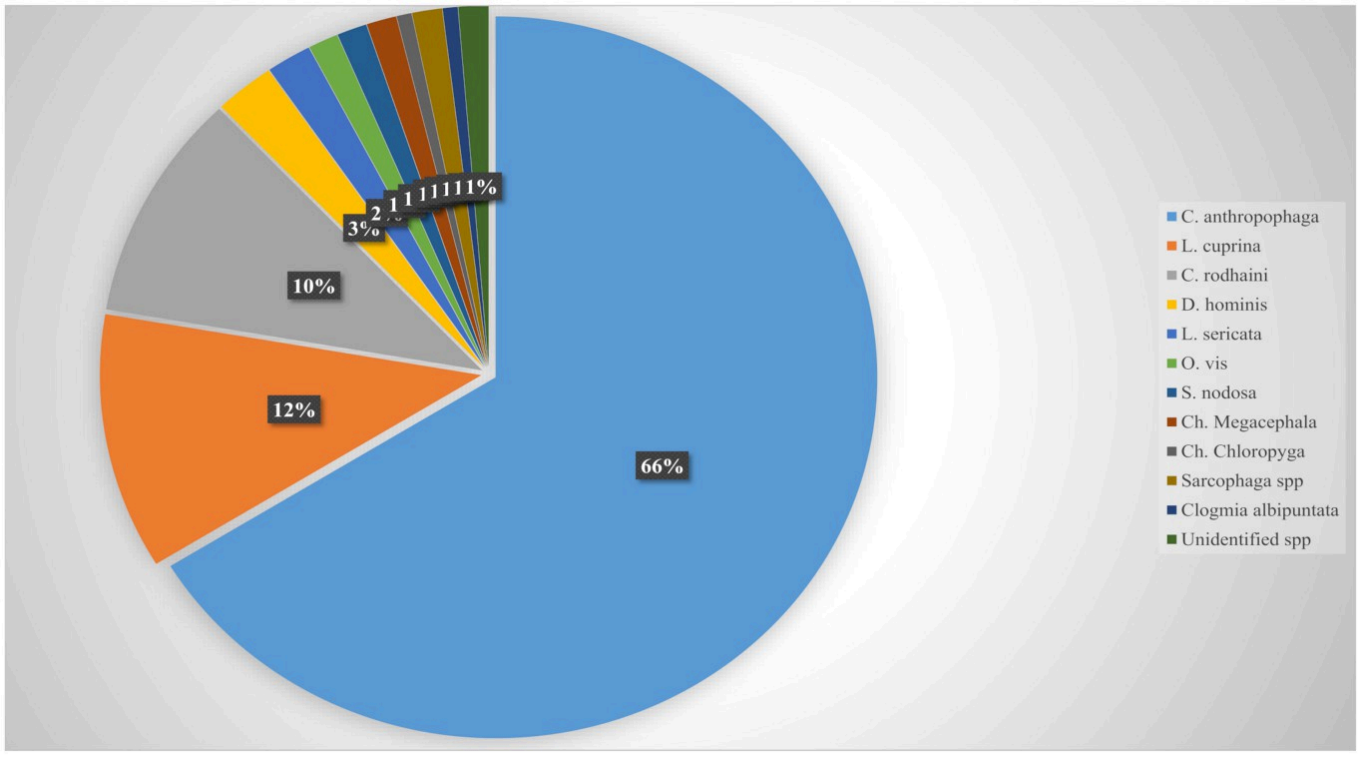
Diversity and prevalence of human myiasis–causing fly species.

Based on reviewed cases, a total of 11 fly species were found to cause human myiasis in SSA which included *Cordylobia anthropophaga*, *Cordylobia rodhaini*, *Dermatobia hominis*, *Lucilia cuprina*, *Lucilia sericata*, *Oestrus ovis*, *Sarcophaga* spp., *Sarcophaga nodosa*, *Chrysomya megacephala*, *Chrysomya chloropyga* and *Clogmia albipuntum*. The obligatory cutaneous parasite *C*. *anthropophaga*, was the most commonly encountered fly species (n = 104), which accounted for 66% of the total cases. *Lucilia cuprina*, which is usually responsible for facultative myiasis, recorded 18 cases which accounts for 11% of the total cases. There were 16 cases of Cordylobia *rodhaini* which accounted for 10% of the cases detected. These three fly species were responsible for the majority of the human myiasis cases in SSA. The other species detected during this study accounted for either 2% or 1% and accumulated to 12% of the total reported cases. The prevalence (percentage) of all the myiasis-causing flies from this study are shown in Fig 3.

Anatomically, the most prevalent sites of infestation were from the lower abdomen down to the lower limbs (abdomen, genital regions, buttocks, both lower and upper thighs, both lower and upper legs, feet). The second most frequent infection sites found in this study were the thoracic and back region (thorax, chest, both shoulders, both breasts, both lower and upper arms including wrists, and upper back). However, the least common infested sites in this study were the head and neck regions (scalp, parietal and temporal lobes, intranasal lesions, forehead, chin, both lower and upper eyelids, truck, nose, maxilla). Three (n = 3) of the studies indicated unspecified infestation sites (various both parts).

Regarding patients’ gender, 78 out of 157 patients were female (49.6%). Males accounted for 38.9% (n = 61) of the studies, and 11.5% (n = 18) were un-identified gender. There were 35 (22.3%) cases reported in SSA with no travel history outside of this area. While 122 (77.7%) of the cases were infected in SSA but reported from countries out of SSA.

## 4. Discussion

Human myiasis is a dermatological condition that can affect people from different areas. However, the most common underlying factor for human myiasis infestation in this study is international travel to endemic regions in SSA [42] [Table 1]. The cases infected and reported in SSA are those patients who are inhabitants (natives) of SSA, and were infected and diagnosed in SSA without traveling out of SSA. While the cases reported outside SSA but are infected in SSA are travelers who visited SSA and got infected in SSA. When they returned to their countries (outside SSA) and got diagnosed, the cases were reported from their countries. With the increase in international travel, there is a higher risk of re-introducing some of these myiasis-causing flies into non-endemic regions of the world that were successful in eradicating these flies [93].

### 4.1 Human myiasis from travelers visiting SSA

In this study, cases were reported from different continents of the world ranging from Europe (mainly Italy) [81], North America (mainly USA) [13,62,79], Asia [20,52,61], North Africa [36–38] to Australia [49] however, North Africa was not included as part of SSA. More than half of the reported cases in this study came from Europe (mainly Italy) [28,40]. This could be because more European travelers visited SSA, also clinicians in these countries report more human myiasis cases due to the availability of entomologist compared to other regions of the world, especially in SSA. While in SSA, only a few countries like Nigeria, Ghana, and South Africa seem to have entomologists [71,74,91].

### 4.2 Reported myiasis causing-species in SSA

*Cordylobia anthropophaga* was the most common myiasis-causing fly species in SSA mainly in West Africa and this information is consistent with previously published literature [52] and the geo-climatic condition in SSA is a suitable breeding ground for this fly (*Cordylobia anthropophaga*). *Cordylobia rodhaini* was most prevalent in East Africa than other parts of SSA [42] however, this species have also been recorded from other parts of SSA which suggest that this fly can survive in different region of the world. The results show that *L*. *cuprina* was most prevalent in Southern Africa. This could be because most of the published literature is from South Africa, and or because the cold weather in some parts of Southern Africa (especially South Africa) is a favourable condition for the breeding of this particular fly species [91]. Although the human botfly (*D*. *hominis*) is not endemic in SSA, there were reported cases of *D*. *hominis* infestation with no travel history to an endemic region (e.g., Latin America) [38,71,72,78]. Although, there is no evidence for this proposition, this phenomenon could be due to climatic changes in SSA, studies on the temperature tolerance, epidemiology, and occurrence pattern of the botfly (*D*. *hominis*) in SSA should be explored to establish the cause for this phenomenon. To enable proper dipteran fly larvae studies, there is a need to extract, identify, and preserve these larvae. Larvae are usually identified morphologically, but sometimes molecular identification is used. Preserving the larvae is very important, especially for molecular identification. Improper preservation can result in DNA degradation that can affect molecular analysis. For morphological identification, larvae are usually killed in warm water of >80°C for 30 seconds to avoid larval decay. Larvae could also be immersed in normal saline or fix in 10% formaldehyde for morphological identification [8,20]. For molecular identification, larvae cannot be killed in hot water. Instead, larvae could be stored in 70%-95% ethanol for a short period or samples can be frozen to -20°C or -80°C for long-term preservation [9,23,44].

### 4.3 Reported myiasis type in SSA

The most common clinical manifestation of myiasis in humans is cutaneous myiasis and this is consistent with the results of this study [11]. As the larvae of *Cordylobia* species are laid in wet environments, they can invade exposed parts of the body (limbs, buttocks, genitals) and subsequently more of these areas would be infected [42]. Several fly species have been found to cause cutaneous myiasis in our study. Different parts of the body (ocular, genitals, nasopharyngeal) could be infested by different fly species resulting in different clinical presentations.

### 4.4 Common identification and treatment (removal) for human myiasis

Human myiasis is usually unpleasant to both patients and health workers, and it is not generally fatal. However, the disease could be serious when it involves delicate parts of the body especially the scalp of young children [6]. Treatment normally involves the extraction of larvae and sometimes the use of antibiotics which has been evident in our study. There are three main larvae removal methods (manual, mechanical, and surgical extraction) described in previous studies and all these extraction methods have been encountered in our study. Manual extraction is usually done with a gentle press on the furuncle with hands (fingers) and paraffin or ointments are usually applied on the central punctum to suffocate the larvae. Dehecq and colleauges [34] manually extracted two larvae from a 9-month-old boy and the lesions healed shortly after extraction [23,66]. There was 45.3% of the reviewed literature which used manual extraction to successfully remove larvae [9,17,30,33,36,37]. None of these cases reported larval fragmentation during the extraction process. In cases involving cutaneous tissues, larvae could be mechanically removed by using specialized devices to aid in larvae extraction. Only 12% of cases in this research used mechanical extraction. Pezzi M and colleauges [8] mechanically extracted 15 larvae from a 45-year-old man, however, 5 of the larvae were damaged. The patient was given a treatment of doxycycline for a week and recovered later [22,66,73]. Of the reported cases, 28% did not specify the extraction method used. Human myiasis can be very severe especially when subcutaneous tissues of the eyes, nose, ears, genitals, and scalp regions of the body are infested [94]. In such extreme cases involving delicate parts of the body, a surgical incision under local anaesthesia (lidocaine) is used to remove larvae. Georgalas I and colleauges [85] used surgical incision to remove a larva from the sub retinal space from a 27-year-old female. Two months after removal, the patient was asymptomatic with visual acuity (VA) of 6/6. Out of the reported cases, 14.6% used surgical extraction to successfully remove larvae and all patients recovered well after incision [35,46,70,74,83].

When larvae are very difficult to detect in subcutaneous tissues, ultrasonography (USG) can be used to detect larvae by scanning the infested area. In such cases, surgical extraction could be used to remove larvae because when other extraction methods are used [83], larvae fragmentation can occur and it could lead to secondary infection or tetanus. Punch biopsy is also an uncommon but very effective method used for larvae detection in subcutaneous lesions [33,64]. Fundoscopy can be used to detect larvae especially with ocular myiasis, and larvae can also be removed through surgical incision [83,85]. Ultrasonography (USG), Punch biopsy, and Fundoscopy were all used in our study to detect larvae in subcutaneous tissues and procedures were successful. These procedures are recorded for use in patients with subcutaneous infestation.

There is no published literature suggesting that sex is a predisposing factor to human myiasis infestation. However, our results have shown that there were more females infected with the disease than males. This could be because more females reported to the hospital when they are sick. Observational and cross-sectional studies should be conducted to ascertain this proposition.

## 5. Limitations

Some reported cases (published papers) of human myiasis from SSA could not be accessed due to lack of institutional access. Therefore, we conclude that the prevalence of human myiasis in SSA could be slightly under-reported.

## 6. Conclusion

Human myiasis is a parasitological condition, which is under-reported and neglected in SSA. Therefore, the actual prevalence and epidemiology of this parasitic infestation is not enough to be estimated. Cases of *D*. *hominis* infestation in SSA with no travel history to Latin America have been reported, therefore, research to study the underlying reasons why this species is resurfacing in SSA should be explored. The molecular identification method should be extensively utilized to identify myiasis-causing flies for its importance in determining myiasis species diversity and epidemic studies. There should be research done to explore dipteran flies’ endemicity in SSA; and the relationship between their prevalence and climatic conditions in the sub-region. Both travelers and natives of SSA (tourists, business people, etc.) should be notified, and provided with adequate information about the prevention of human myiasis at entry points and at a community level in endemic regions. There is also a need to raise awareness for inhabitants, clinicians, and travelers to these regions about some of the symptoms and predisposing factors for human myiasis.

## Supporting information

S1 Dataset Metadata of the study. **Sheet A**-Number and Percentage of human myiasis-causing fly species. **Sheet B**-Number and percentage of the Sex of infected patients. **Sheet C-**Number and percentage of human myiasis types. **Sheet D-** Summary of selected studies.
(XLSX)

S1 Table Risk of Bias of the Selected Studies by JBI-MAStARI. The items were collapsed into 8 quality-appraisal criteria (Q1-Were patient’s demographic characteristics clearly described? Q2-Was the patient’s history clearly described and presented as a timeline? Q3-Was the current clinical condition of the patient on presentation clearly described? Q4-Were diagnostic tests or assessment methods and the results clearly described? Q5-Was the intervention(s) or treatment procedure(s) clearly described? Q6-Was the post-intervention clinical condition clearly described? Q7-Were adverse events (harms) or unanticipated events identified and described? Q8-Does the case report provide takeaway lessons?). JBI-MAStARI was used to assess risk of bias. Articles that scored between 1 and 2 were classified as low methodological quality, articles with scores between 3 and 4 were classified as moderate quality, and those with scores ≥ 5 were classified as high quality. N: no, NA: not applicable, U: unclear, Y: yes.
(DOCX)

S2 Table PRISMA checklist. (DOCX)
