# Supplementary material for: Human myiasis in Sub-Saharan Africa: A systematic review
Source: PLoS Negl Trop Dis. 2024 Mar 28;18(3):e0012027. doi: 10.1371/journal.pntd.0012027 (PMC10977789; doi:10.1371/journal.pntd.0012027)
Supplement: S1 Table — The items were collapsed into 8 quality-appraisal criteria (Q1-Were patient’s demographic characteristics clearly described? Q2-Was the patient’s history clearly described and presented as a timeline? Q3-Was the current clinical condition of the patient on presentation clearly described? Q4-Were diagnostic tests or assessment methods and the results clearly described? Q5-Was the intervention(s) or treatment procedure(s) clearly described? Q6-Was the post-intervention clinical condition clearly described? Q7-Were adverse events (harms) or unanticipated events identified and described? Q8-Does the case report provide takeaway lessons?). JBI-MAStARI was used to assess risk of bias. Articles that scored between 1 and 2 were classified as low methodological quality, articles with scores between 3 and 4 were classified as moderate quality, and those with scores ≥ 5 were classified as high quality. N: no, NA: not applicable, U: unclear, Y: yes. (DOCX) [file pntd.0012027.s002.docx]

***S1 Table: Risk of Bias of the Selected Studies by JBI-MAStARI***

| **Supplementary Annex S1:** Risk of Bias of the Selected Studies by JBI-MAStARI | | | | | | | | | |
| --- | --- | --- | --- | --- | --- | --- | --- | --- | --- |
| **References** | **Q1** | **Q2** | **Q3** | **Q4** | **Q5** | **Q6** | **Q7** | **Q8** | **Yes, Score (_/8)**  **Methodological quality** |
| ***Central Africa*** |  |  |  |  |  |  |  |  |  |
| [1] | Y | Y | Y | Y | Y | Y | Y | Y | 8/8 |
| [2] | Y | Y | Y | Y | Y | Y | Y | Y | 8/8 |
| [3] | Y | Y | Y | Y | Y | Y | Y | Y | 8/8 |
| [4] | Y | Y | Y | Y | Y | Y | Y | Y | 8/8 |
| [5] | Y | Y | Y | Y | Y | Y | Y | Y | 8/8 |
| [6] | Y | Y | Y | Y | Y | Y | Y | Y | 8/8 |
| [7] | Y | Y | Y | Y | Y | Y | Y | Y | 8/8 |
| [8] | Y | Y | Y | Y | Y | N | N | N | 5/8 |
| [9] | Y | Y | Y | Y | Y | Y | Y | Y | 8/8 |
| [10] | Y | Y | Y | Y | Y | N | N | Y | 6/8 |
| [11] | Y | Y | Y | Y | Y | Y | Y | Y | 8/8 |
| [12] | Y | Y | Y | Y | Y | Y | Y | Y | 8/8 |
| [13] | Y | N | Y | Y | Y | N | N | Y | 5/8 |
| ***East Africa*** |  |  |  |  |  |  |  |  |  |
| [14] | Y | Y | Y | Y | N | N | N | N | 4/8 |
| [15] | Y | Y | Y | Y | Y | Y | Y | Y | 8/8 |
| [16] | Y | Y | Y | Y | Y | Y | Y | Y | 8/8 |
| [17] | Y | Y | Y | Y | Y | Y | Y | Y | 8/8 |
| [18] | Y | Y | Y | Y | Y | Y | Y | Y | 8/8 |
| [19] | Y | Y | Y | Y | Y | Y | Y | Y | 8/8 |
| [20] | Y | Y | Y | Y | Y | Y | Y | Y | 8/8 |
| [21] | Y | Y | Y | Y | Y | Y | Y | Y | 8/8 |
| [22] | Y | Y | Y | Y | Y | Y | Y | Y | 8/8 |
| [23] | Y | Y | Y | Y | Y | Y | Y | Y | 8/8 |
| [24] | Y | Y | Y | Y | Y | Y | Y | Y | 8/8 |
| [25] | Y | Y | Y | Y | Y | Y | Y | Y | 8/8 |
| [26] | Y | Y | Y | Y | Y | N | N | Y | 6/8 |
| [27] | Y | Y | Y | Y | Y | Y | Y | Y | 8/8 |
| [28] | Y | Y | Y | Y | Y | Y | Y | Y | 8/8 |
| [29] | Y | Y | Y | Y | Y | Y | Y | Y | 8/8 |
| [30] | Y | Y | Y | Y | Y | Y | Y | Y | 8/8 |
| [31] | Y | Y | Y | Y | Y | Y | Y | Y | 8/8 |
| [32] | Y | Y | Y | Y | Y | Y | Y | Y | 8/8 |
| [33] | Y | Y | Y | Y | Y | Y | Y | Y | 8/8 |
| [34] | Y | Y | Y | Y | Y | Y | Y | Y | 8/8 |
| [35] | Y | Y | Y | Y | Y | Y | Y | Y | 8/8 |
| [36] | Y | Y | Y | Y | Y | Y | Y | Y | 8/8 |
| [15] | Y | Y | Y | Y | Y | Y | Y | Y | 8/8 |
| ***West Africa*** |  |  |  |  |  |  |  |  |  |
| [37] | Y | Y | Y | Y | Y | Y | Y | Y | 8/8 |
| [38] | Y | Y | Y | Y | Y | Y | Y | Y | 8/8 |
| [2] | Y | Y | Y | Y | Y | Y | Y | Y | 8/8 |
| [39] | Y | Y | Y | Y | Y | Y | Y | Y | 8/8 |
| [40] | Y | Y | Y | Y | Y | Y | Y | Y | 8/8 |
| [41] | Y | Y | Y | Y | Y | Y | Y | Y | 8/8 |
| [42] | Y | Y | Y | Y | Y | Y | Y | Y | 8/8 |
| [43] | Y | Y | Y | Y | Y | Y | Y | Y | 8/8 |
| [44] | Y | Y | Y | Y | Y | Y | Y | Y | 8/8 |
| [45] | Y | Y | Y | Y | Y | Y | N | Y | 8/8 |
| [46] | Y | Y | Y | Y | Y | Y | N | Y | 8/8 |
| [47] | Y | Y | Y | Y | Y | Y | Y | Y | 8/8 |
| [48] | Y | Y | Y | Y | Y | Y | Y | Y | 8/8 |
| [49] | Y | Y | Y | Y | Y | Y | N | Y | 7/8 |
| [50] | Y | Y | Y | Y | Y | Y | N | Y | 7/8 |
| [51] | Y | Y | Y | Y | Y | Y | Y | Y | 8/8 |
| [52] | Y | Y | Y | Y | Y | N | N | Y | 6/8 |
| [53] | Y | Y | Y | Y | Y | Y | Y | Y | 8/8 |
| [54] | Y | Y | Y | Y | Y | Y | N | Y | 7/8 |
| [55] | Y | Y | Y | Y | Y | Y | Y | Y | 8/8 |
| [56] | Y | Y | Y | Y | Y | Y | Y | Y | 8/8 |
| [57] | Y | Y | Y | Y | Y | N | N | Y | 6/8 |
| [58] | Y | Y | N | N | N | N | N | N | 2/6 |
| [59] | Y | Y | Y | Y | Y | Y | Y | Y | 8/8 |
| [60] | Y | Y | Y | Y | Y | Y | Y | Y | 8/8 |
| [61] | Y | Y | Y | Y | Y | Y | Y | Y | 8/8 |
| [62] | Y | Y | Y | Y | Y | Y | Y | Y | 8/8 |
| [63] | Y | Y | Y | Y | Y | Y | N | Y | 7/8 |
| [64] | Y | Y | Y | Y | Y | Y | Y | Y | 8/8 |
| ***Southern Africa*** |  |  |  |  |  |  |  |  |  |
| [65] | Y | Y | Y | Y | Y | Y | N | Y | 7/8 |
| [66] | Y | Y | Y | Y | Y | Y | Y | Y | 8/8 |
| [67] | Y | Y | Y | Y | Y | Y | N | Y | 7/8 |
| [68] | Y | Y | Y | Y | Y | Y | Y | Y | 8/8 |
| [69] | Y | Y | Y | Y | Y | Y | Y | Y | 8/8 |
| [70] | Y | Y | Y | Y | Y | Y | N | Y | 7/8 |
| [71] | Y | Y | Y | Y | Y | Y | Y | Y | 8/8 |
| [72] | Y | Y | Y | Y | Y | Y | N | Y | 7/8 |
| [73] | Y | Y | Y | Y | Y | Y | N | Y | 7/8 |
| [74] | Y | Y | Y | Y | Y | Y | Y | Y | 8/8 |
| [75] | Y | Y | Y | Y | Y | Y | Y | Y | 8/8 |

The items were collapsed into 8 quality-appraisal criteria (Q1-Were patient’s demographic characteristics clearly described? Q2-Was the patient’s history clearly described and presented as a timeline? Q3-Was the current clinical condition of the patient on presentation clearly described? Q4-Were diagnostic tests or assessment methods and the results clearly described? Q5-Was the intervention(s) or treatment procedure(s) clearly described? Q6-Was the post-intervention clinical condition clearly described? Q7-Were adverse events (harms) or unanticipated events identified and described? Q8-Does the case report provide takeaway lessons?).

JBI-MAStARI was used to assess risk of bias. Articles that scored between **1 and 2** were classified as **low methodological quality**, articles with scores between **3 and 4** were classified as **moderate quality**, and those with **scores ≥ 5** were classified as **high quality**.

N: no, NA: not applicable, U: unclear, Y: yes.

REFERENCES

1. Pathania V, Kashif AW, Aggarwal RN. Cutaneous myiasis: Think beyond furunculosis. Medical Journal Armed Forces India. 2018;74(3):268-72.

2. Blaizot R, Vanhecke C, Le Gall P, Duvignaud A, Receveur MC, Malvy D. Furuncular myiasis for the Western dermatologist: treatment in outpatient consultation. Int J Dermatol. 2018;57(2):227-30.

3. Schubert L, Tobudic S, Sillaber C, Winkler S. The swollen lip: unusual presentation of furuncular myiasis in a returning traveller. J Travel Med. 2022;29(5).

4. Suárez JA, Ying A, Orillac LA, Cedeño I, Sosa N. First case of Furuncular Myiasis due to Cordylobia anthropophaga in a Latin American resident returning from Central African Republic. Braz J Infect Dis. 2018;22(1):70-3.

5. Ko JY, Lee IY, Park BJ, Shin JM, Ryu JS. A Case of Cutaneous Myiasis Caused by Cordylobia anthropophaga Larvae in a Korean Traveler Returning from Central Africa. Korean J Parasitol. 2018;56(2):199-203.

6. Naotunna TdS, Ismail M, Ihalamulla R. The second case of cutaneous myiasis caused by Cordylobia anthropophaga (Tumbu fly) in Sri Lanka. 2000.

7. Hakeem MJML, Bhattacharyya DN. Exotic human myiasis. Travel Medicine and Infectious Disease. 2009;7(4):198-202.

8. Koźmińska-Kubarska A. Cordylobia anthropophaga infestation. Int J Dermatol. 1981;20(7):495-6.

9. Dehecq E, Nzungu PN, Cailliez JC, Guevart E, Delhaes L, Dei-Cas E, et al. Cordylobia anthropophaga (Diptera: Calliphoridae) outside Africa: a case of furuncular myiasis in a child returning from Congo. J Med Entomol. 2005;42(2):187-92.

10. Pica R, Castellano C, Pignata D, Ipri D. Human cutaneous myiasis: a case report. La Clinica Terapeutica. 2008;159(6):431-3.

11. Ajili F, Abid R, Bousseta N, Mrabet A, Karoui G, Louzir B, et al. [Antibiotic resistant furuncles: think myiasis]. Pan Afr Med J. 2013;15:41.

12. Vanhecke C, Nguimfack RN, Lemarchand J, Reichart V, Le Gall P. [Facial edema caused by multifocal myiasis of Cordylobia rodhaini in Yaounde - Cameroon]. Presse Med. 2015;44(5):564-6.

13. Frikh R, Hjira N, Frikh M, Baba N, Ghfir M, Lmimouni B, et al. Furuncular myiasis: unusual case of African Dermatobia hominis. Dermatol Online J. 2009;15(9):11.

14. Rotte M, Fields M. That's Not An Abscess! Furuncular myiasis. Ann Emerg Med. 2013;62(1):98, 103.

15. James AS, Stevenson J. Cutaneous myiasis due to Tumbu fly. Arch Emerg Med. 1992;9(1):58-61.

16. Hasegawa M, Harada T, Kojima Y, Nakamura A, Yamada Y, Kadosaka T, et al. An imported case of furuncular myiasis due to Cordylobia anthropophaga which emerged in Japan. British Journal of Dermatology. 2000;143(4):912-4.

17. Musa HA, Allah EW. Cutaneous myiasis caused by Cordylobia Anthropophaga: description of a case from Gazira State–Sudan. Sudanese J Pub Health. 2008;3(2):91-3.

18. Dires A, Kebede A, Gedamu S, Dires T. Case of multiple furuncular myiasis in Northeast Ethiopia. Clin Case Rep. 2022;10(7):e6015.

19. Veraldi S, Serini SM, Süss L. Three cases of cutaneous myiasis caused by Cordylobia rodhaini. J Infect Dev Ctries. 2014;8(2):249-51.

20. Tolera TB. Human cutaneous myiasis under-reported in Dilla, Ethiopia. Journal of Clinical & Medical Case Reports. 2017;3(23):1-5.

21. Oliva E, Bargiggia G, Quinzan G, Lanza P, Farina C. Furuncular myiasis in Italian traveler returning from Kenya. J Infect Dev Ctries. 2020;14(1):114-6.

22. Yasukawa K, Dass K. Myiasis due to Cordylobia anthropophaga. Am J Trop Med Hyg. 2020;102(2):251.

23. Sivelli P, Vinciguerra R, Tondini L, Cavalli E, Galli A, Chelazzi P, et al. Eyelid myiasis caused by Cordylobia anthropophaga. Ocular Immunology and Inflammation. 2015;23(3):259-60.

24. Pezzi M, Cultrera R, Chicca M, Leis M. Furuncular Myiasis Caused by Cordylobia rodhaini (Diptera: Calliphoridae): A Case Report and a Literature Review. J Med Entomol. 2015;52(2):151-5.

25. Song SM, Kim SW, Goo YK, Hong Y, Ock M, Cha HJ, et al. A Case of Furuncular Myiasis Due to Cordylobia anthropophaga in a Korean Traveler Returning from Uganda. Korean J Parasitol. 2017;55(3):327-31.

26. Novati S, Sacchi L, Chichino G, Scaglia M. [Furuncular myiasis caused by Cordylobia anthropophaga: description of a case from Tanzania]. Parassitologia. 1994;36(3):265-7.

27. Parkhouse D. Cutaneous myiasis due to the Tumbu fly during Operation Keeling. J R Army Med Corps. 2004;150(1):24-6.

28. Geary MJ, Russell RC, Hudson BJ, Hardy A. Exotic myiasis with Lund's fly (Cordylobia rodhaini). Medical journal of Australia. 1999;171(11-12):654-5.

29. Pampiglione S, Schiavon S, Candiani G, Fioravanti ML. [Clinical and parasitological observations on a case of disseminated furuncular myiasis caused by Cordylobia rodhaini in a man in Ethiopia]. Parassitologia. 1991;33(2-3):159-67.

30. Strohbücker L, Dissemond J, Körber A. [Inflammatory papules and nodi in a 52-year-old woman after a vacation in Zanzibar]. Hautarzt. 2016;67(8):667-9.

31. Deng Y, Liu F, Chen X, Lu S. The first imported cutaneous myiasis due to Cordylobia anthropophaga in China. Int J Dermatol. 2013;52(1):120-2.

32. Wade N, Shahi F, Mawer D, Brown N. Rare cutaneous myiasis of the face due to Lund's fly (Cordylobia rodhaini) in a British traveller returning from Uganda. BMJ Case Rep. 2019;12(1).

33. Ruan W, Feng Y, Zhang L, Sun J, Yao L. Health problems associated with international travel: a case of cutaneous myiasis in China due to Cordylobia anthropophaga imported from Uganda. Biosci Trends. 2014;8(6):346-9.

34. Hannam P, Khairnar K, Downey J, Powis J, Ralevski F, Pillai DR. Cutaneous myiasis in traveler returning from Ethiopia. Emerg Infect Dis. 2011;17(12):2385-6.

35. Roberts LW, Boyce WL, Lyerly WH, Jr. Cordylobia anthropophaga (Diptera: Calliphoridae) myiasis in an infant and dog and a technique for larval rearing. J Med Entomol. 1982;19(3):350-1.

36. Palmieri JR, North D, Santo A. Furuncular myiasis of the foot caused by the tumbu fly, Cordylobia anthropophaga: report in a medical student returning from a medical mission trip to Tanzania. Int Med Case Rep J. 2013;6:25-8.

37. Ogbalu OK, Achufusi TGO, Orlu EE. Epidemiology of human furuncular myiasis of Cordylobia anthropophaga (Grunberg) in Nigeria. International Journal of Dermatology. 2013;52(3):331-6.

38. Grassi V, Butterworth JW, Latiffi L. Cordylobia rodhaini infestation of the breast: Report of a case mimicking a breast abscess. Int J Surg Case Rep. 2016;27:122-4.

39. Petersen C, Zachariae C. Acute balanoposthitis caused by infestation with Cordylobia anthropophaga. Acta dermato-venereologica. 1999;79(2).

40. Ogbalu OK, Achufusi TGO, Adibe C. Incidence of multiple myiases in breasts of rural women and oral infection in infants from the human warble fly larvae in the humid Tropic‐Niger Delta. International journal of dermatology. 2006;45(9):1069-70.

41. Sharma P, Pai HS, Pai GS. Furuncular myiasis mimicking pyoderma. 2008.

42. Schechter E, Lazar J, Nix ME, Mallon WK, Moore CL. Identification of subcutaneous myiasis using bedside emergency physician performed ultrasound. The Journal of emergency medicine. 2011;40(1):e1-e3.

43. Tamir J, Haik J, Schwartz E. Myiasis with Lund's fly (Cordylobia rodhaini) in travelers. J Travel Med. 2003;10(5):293-5.

44. Rimoin L, Jackson J, Yang A, Goh C, Soriano T. Furuncular myiasis in 2 American travelers returning from Senegal. Cutis. 2014;94(6):281-4.

45. Logar J, Soba B, Parac Z. Cutaneous myiasis caused by Cordylobia anthropophaga. Wien Klin Wochenschr. 2006;118(5-6):180-2.

46. How EH, Yap D, Mbakada N. An exotic abscess within the United Kingdom from The Gambia: a case report. J Med Case Rep. 2017;11(1):310.

47. Lodi A, Bruscagin C, Gianni C, Mancini LL, Crosti C. Myiasis due to Cordylobia anthropophaga (Tumbu-fly). Int J Dermatol. 1994;33(2):127-8.

48. Bardach H, Aspöck H. [Furunculoid myiasis due to Cordylobia anthropophaga in a traveler returning from Africa and review of the literature]. Z Hautkr. 1981;56(4):216-20.

49. Onyeama C, Njai P. Cutaneous myiasis (Tumbu fly larvae): A case report. Nigerian Journal of Paediatrics. 2005;32(1):26-7.

50. Kovaleva A, Climent PC, Bécares CV, Martín Azaña MJ, Irishina N, Goy EI. Urogenital myiasis by Cordylobia anthropophaga. J Pediatr Adolesc Gynecol. 2013;26(6):e123-5.

51. Fusco FM, Nardiello S, Brancaccio G, Rossiello R, Gaeta GB. [Cutaneous myiasis from Cordylobia anthropophaga in a traveller returning from Senegal: a case study]. Infez Med. 2005;13(2):109-11.

52. Nwosu PU, Dakul DA. Report of a case of cutaneous (furuncular) and gastrointestinal myiasis (dermatobia hominis) in a Nigerian child. West Afr J Med. 2013;32(2):149-52.

53. Rodríguez-Cerdeira C, Gregorio MC, Guzman RA. Dermatobia Hominis Infestation Misdiagnosed as Abscesses in a Traveler to Spain. Acta Dermatovenerol Croat. 2018;26(3):267-9.

54. Devambez H, Richeux M, Guericolas M, Choquet C, Casalino E, Ghazali AD. Eyelid inflammation: An uncommon cause in occidental countries. Am J Emerg Med. 2017;35(11):1789.e3-.e5.

55. Choontanom R, Thanos S, Busse H, Stupp T. A souvenir from Ghana. J Pediatr. 2008;153(2):297.

56. Wangia M, Glenn C, Mitchell C, Fisher S. Florid Cordylobia anthropophaga furuncular myiasis from travel in Nigeria. J Dermatol. 2012;39(12):1099-100.

57. Mohammed N, Smith KG. Letter: Nasopharyngeal myiasis in man caused by larve of Clogmia (=Telmetoscopus) albipunctatus Williston (Psychodidae, Dipt.). Trans R Soc Trop Med Hyg. 1976;70(1):91.

58. Schouten W, Kager P. Diagnostic image (109). A man with furuncles. Cutaneous myiasis. Nederlands tijdschrift voor geneeskunde. 2002;146(41):1937.

59. Ogunniyi IO. Furuncular swelling caused by larva of Cordylobia anthropophaga in Kaduna, Nigeria. Trans R Soc Trop Med Hyg. 1981;75(5):752.

60. Messahel A, Sen P, Wilson A, Patel M. An unusual case of myiasis. Journal of Infection and Public Health. 2010;3(1):43-5.

61. Malek AE, Ostrosky-Zeichner L. Furuncular myiasis in a traveller to West Africa. J Travel Med. 2021;28(6).

62. Adisa CA, Mbanaso A. 'Furuncular myiasis of the breast caused by the larvae of the Tumbu fly (Cordylobia anthropophaga)'. BMC Surg. 2004;4:5.

63. Veraldi S, Brusasco A, Süss L. Cutaneous myiasis caused by larvae of Cordylobia anthropophaga (Blanchard). Int J Dermatol. 1993;32(3):184-7.

64. Ogbalu OK, Achufusi TG, Adibe C. Incidence of multiple myiases in breasts of rural women and oral infection in infants from the human warble fly larvae in the humid Tropic-Niger Delta. Int J Dermatol. 2006;45(9):1069-70.

65. Kuria S, Kingu H, Vasaikar S, Mkhize J, Iisa J, Dhaffala A. New fly species causing human myiasis identified in Eastern Cape, South Africa. SAMJ: South African Medical Journal. 2010;100(9):580-1.

66. Parikh V, Biswas J, Vaijayanthi K, Das D, Raval V. Bilateral ocular myiasis interna caused by botfly (Oestrus ovis): a case report. Ocular Immunology and Inflammation. 2011;19(6):444-7.

67. Van Niekerk G, Henning M, Coetzee M. Outbreak of myiasis. South African Medical Journal. 2007;97(2):112-4.

68. Georgalas I, Ladas I, Maselos S, Lymperopoulos K, Markomichelakis N. Intraocular safari: ophthalmomyiasis interna. Clinical & experimental ophthalmology. 2011;39(1):84-5.

69. Musaya J, Mponda K. Case Report: Furuncular Myiasis in Malawi. Wellcome Open Research. 2020;5(41):41.

70. Lee E, Robinson F. Furuncular myiasis of the face caused by larva of the Tumbu fly (Cordylobia anthropophaga). Eye. 2007;21(2):268-9.

71. Kingu HJ, Kuria SK, Villet MH, Mkhize JN, Dhaffala A, Iisa JM. Cutaneous myiasis: is Lucilia cuprina safe and acceptable for maggot debridement therapy? 2012.

72. Ng SOC, Yates M. Cutaneous myiasis in a traveller returning from Africa. Australasian journal of dermatology. 1997;38(1):38-9.

73. Weightman NC, Mitra S, Kipling PT. Clinical microbiological case: Itchy furunculosis on return from South Africa. Clin Microbiol Infect. 2003;9(12):1249, 67-8.

74. Lowe P, Naseem S, Bailey C. Cordylobia anthropophaga: a rare surgical emergency in the UK. BMJ Case Rep. 2013;2013.

75. Kuria SK, Kingu HJ, Villet MH, Dhaffala A. Human myiasis in rural South Africa is under-reported. S Afr Med J. 2015;105(2):129-33.
